# Neural systems mediating processing of sound units of language distinguish recovery versus persistence in stuttering

**DOI:** 10.1186/s11689-015-9124-7

**Published:** 2015-08-18

**Authors:** Ranjini Mohan, Christine Weber

**Affiliations:** Department of Speech Language and Hearing Science, Purdue University, Lyles Porter Hall, 715 Clinic Drive, West Lafayette, IN USA

**Keywords:** Stuttering persistence, Phonological processing, N400, Rhyme effect, Lateralization

## Abstract

**Background:**

Developmental stuttering is a multi-factorial disorder. Measures of neural activity while children processed the phonological (language sound unit) properties of words have revealed neurodevelopmental differences between fluent children and those who stutter. However, there is limited evidence to show whether the neural bases of phonological processing can be used to identify stuttering recovery status. As an initial step, we aimed to determine if differences in neural activity during phonological processing could aid in distinguishing children who had recovered from stuttering and those whose stuttering persisted.

**Methods:**

We examined neural activity mediating phonological processing in forty-three 7-8 year old children. Groups included children who had recovered from stuttering (CWS-Rec), those whose stuttering persisted (CWS-Per), and children who did not stutter (CWNS). All children demonstrated normal non-verbal intelligence and language skills. Electroencephalograms were recorded as the children listened to pairs of pseudo-words (primes-targets) that either rhymed or did not. Behavioral rhyme judgments along with peak latency and mean amplitude of the N400s elicited by prime and target stimuli were examined.

**Results:**

All the groups were very accurate in their rhyme judgments and displayed a typical ERP rhyme effect, characterized by increased N400 amplitudes over central parietal sites for nonrhyming targets compared to rhyming targets. However, over anterior electrode sites, an earlier onset of the N400 for rhyming compared to non-rhyming targets, indexing phonological segmentation and rehearsal, was observed in the CWNS and CWS-Rec groups. This effect occurred bilaterally for the CWNS, was greater over the right hemisphere in the CWS-Rec, and was absent in the CWS-Per.

**Conclusions:**

These results are the first to show that differences in ERPs reflecting phonological processing are marked by atypical lateralization in childhood even after stuttering recovery and more pronounced atypical neural patterns for the children whose stuttering persisted. Despite comparable language and phonological skills as revealed by standardized tests, the neural activity mediating phonological segmentation and rehearsal differentiated 7-8 year old children whose stuttering persisted from those who had recovered from stuttering and typically developing peers.

## Background

Approximately 5–8 % of preschool children display stuttering-like disfluencies characterized by sound and syllable repetitions, prolongations of speech sounds, dysrhythmic phonations, and silent blocks [1]. While up to 80 % of the children who stutter eventually recover, we are unable to predict with a reasonable degree of certainty which children are at high risk for developing persistent stuttering and may benefit most from early intervention. Estimates of stuttering recovery depend, to some extent, on the age of the children at initial assessment. For example, recovery rates are estimated to be approximately 80 % when sampling children near the typical onset of stuttering, i.e., at ages 2–3 years [1, 2], and these fall to approximately 50 % for 4-year-old children [2].

With considerable incidence of stuttering in preschoolers, resources are not available to treat every child who begins to stutter. Often, treatment is delayed for those children who ultimately persist in stuttering. For these at-risk children, early intervention is beneficial for improving fluency as well as for providing educational and emotional support for the child and family [2, 3]. The current limitations in reliably predicting eventual recovery or persistence in stuttering also has implications for research: studies of young children who stutter may contain confounds or increased variability due to the eventual recovery or persistence status of the participants.

In order to address the limitations in early identification of at-risk children, recent studies have focused on identifying behavioral factors that predict the course of stuttering [4–6]. While these studies have diagnostic advantages, stuttering is a highly heterogeneous, neurodevelopmental disorder [7, 8], resulting from atypical interaction of neural regions and connections that support speech and language processes [9, 10]. Although the diagnostic value of neurophysiological measures is currently limited, it may be highly relevant for predicting stuttering chronicity in the future. Therefore, a crucial step towards predicting stuttering persistence and recovery using neurophysiological measures is to evaluate the neurodevelopmental differences between children whose stuttering persists and those who have already recovered from stuttering. Neuroimaging techniques [11] and electrophysiological correlates of language processing [12] have been used to characterize neurodevelopmental differences between stuttering recovery and persistence. In the current study, we compare neurophysiological functions underlying one aspect of language, the processing of sound units of language or phonological processing, for three groups of young school-age children: children who have recovered from stuttering (CWS-Rec), children whose stuttering persists (CWS-Per), and children who did not stutter (CWNS).

### Language abilities in children who stutter

Over recent years, a coherent picture of the essential characteristics of developmental stuttering has emerged, laying the groundwork for identifying the factors that may play a role in recovery or persistence. Evidence from a multitude of studies utilizing a variety of methodological approaches indicates that the development of stuttering involves a variety of factors in the domains of speech motor control, language, and emotion/temperament (for review, please see [1, 2, 7, 13, 14]). For example, a meta-analysis of 22 studies revealed that children who stutter (CWS) exhibited lower mean scores on tests of receptive and expressive vocabulary as well as lower mean length of utterance as compared to their fluent peers, even though they scored within normal limits [15]. A growing body of literature has emphasized neurodevelopmental differences in children who stutter compared to their typically developing peers, including differences in grey matter volumes in speech-related regions, white matter connectivity for speech motor control, and neural correlates of phonological processing [9, 11, 16–21].

It has been reported that phonological disorders occur at a higher rate among the stuttering population relative to the general population [22, 23]. To investigate the phonological proficiency in CWS, researchers have used a number of behavioral tasks, including priming, rhyme judgment, and non-word repetition [21, 24–28]. For example, findings of lower rhyme judgment accuracy [21] and lower accuracy on non-word repetition tests among CWS relative to CWNS [26, 28] indicate an association between phonological proficiency and stuttering. Providing further evidence for the role of phonology in stuttering, Spencer and Weber-Fox [4] found that performance on the Bankson and Bernthal Test of Phonology—consonant inventory subtest (BBTOP-CI; [29]) as well as the Dollaghan and Campbell non-word repetition test [30] was predictive of stuttering persistence in preschool children.

### Neural correlates of phonological processing in children who stutter

Standardized tests of phonology, behavioral measures of phonological encoding and phonological working memory often distinguish CWS and their fluent peers [24, 26–28, 31, 32]. Recognizing the neurodevelopmental nature of stuttering, a few researchers explored the underlying neurophysiological mechanisms of phonological processing. Sato and colleagues [19] used near-infrared spectroscopy (NIRs) to assess cortical auditory speech processing using phonemic and prosodic contrast syllables in adults and children (3–5 years and 6–12 years) who stutter and age-matched controls. The age-matched controls (both adults and children) exhibited increased concentration of hemoglobin in the left hemisphere for phonemic contrasts and in the right hemisphere for prosodic contrasts. The adults and children who stutter, however, did not show a left-lateralized response for the phonemic contrasts, suggesting atypical auditory functional lateralization for phonological processing.

Electrophysiological measures have also provided valuable information about the neurodevelopmental differences in phonological processing in school-age CWS. Weber-Fox et al. [21] compared 9–13-year-old CWS and CWNS on a visual rhyme judgment task, using real-word prime-target pairs. Peak latency of the N400 elicited by target words was earlier over the left hemisphere for CWNS, while it was earlier over the right hemisphere in CWS. This indicated that the relative timing of hemispheric neural processing for the rhyming/non-rhyming stimuli distinguished CWS from CWNS.

Researchers comparing phonological processing in both older school-age children [21] and adults who stutter [33] with their fluent peers showed that for the same task, behavioral and electrophysiological responses were qualitatively different in adults and children who stutter. In a visual rhyme judgment task where orthographic and phonologic congruence was altered, adults who stutter did not differ from their fluent peers in overall accuracy or reaction time for rhyme judgments [33] but 9–12-year-old CWS displayed lower accuracy as compared to their typically fluent peers [21]. Furthermore, compared to CWNS, the CWS displayed reduced mean amplitude of the contingent negative cariation (CNV), an event-related potential (ERP) component that has been thought to index silent rehearsal of the prime [21, 34]. Another interpretation from ERP studies of rhyming in children indicate that the CNV may index resource allocation for phonological processing of both written words [35] and auditorily presented non-words [36].The CNV and N400 elicited for rhyming in adults who stutter were similar to those of their fluent peers. The difference, however, was that the adults who stutter displayed a greater right hemisphere asymmetry of the N400 elicited by the rhyme/non-rhyme targets [33]. Taken together, the two studies indicate that factors such as maturation and experience with stuttering are likely to affect neural functions during the course of development. Because of this, studying phonological processing in younger CWS closer to the age of onset will provide needed information regarding how or if neural networks related to phonological processing play a role during earlier stages of development of the disorder.

### Recovery and persistence of stuttering

Phonological proficiency has also been associated with eventual recovery or persistence in stuttering [1, 2, 4–6, 37]. Converging evidence from a variety of studies indicates that stuttering persistence, on average, is associated with lower phonological and articulation proficiencies compared to those of preschool children who would eventually recover [4, 6]. However, authors from these studies note that there were wide ranges of scores within the groups as well as significant overlap of scores between groups and suggest that additional factors are necessary for reliable prediction. The large variability in behavioral scores across tests of expressive and receptive language [15] as well as tests of phonology [4–6] indicates that differences between children who persist and those recovered from stuttering may be too subtle to be uniformly characterized by standardized tests [13]. Characterizing crucial neurophysiological processes in CWS-Rec and CWS-Per will shed light on possible underlying neurodevelopmental differences between stuttering recovery and persistence.

To date, only one study has assessed potential neuroanatomical differences in speech-related areas among children who had recovered from stuttering and those who persisted. Chang et al. [11] utilized measures of fractional anisotropy to study brain structure differences in CWNS, CWS-Rec, and CWS-Per between the ages of 9 and 12 years. Their findings indicated that white matter tract integrity in left hemisphere speech areas, including the arcuate fasciculus, was reduced in CWS-Per. The arcuate fasciculus connects regions involved in phonological operations such as rhyming [38] and measures of diffusion in the arcuate fasciculus have also been correlated with phonological awareness skills in children 7–11 years old [39]. CWS-Rec, on the other hand, exhibited normal fractional anisotropy in the left hemisphere but a trend towards greater right fractional anisotropy compared to the other two groups. Chang and colleagues concluded that the reduction in white matter tract integrity in regions involved in the speech production network may indicate a risk for persistent stuttering and suggested that differences in neural connectivity may differentiate children who had recovered from those whose stuttering persisted.

### The current study

In the current study, we focus on how underlying brain functions related to phonological processing may be associated with the recovery or persistence of stuttering. We compared the ERPs elicited in a rhyming paradigm for 7–8-year-old CWNS, CWS-Rec, and CWS-Per. Such rhyme judgments are known to rely on phonological awareness [40, 41].

Rhyming paradigms employing ERP measures, such as in our study, have elicited a reliable ERP index, the N400 [21, 35, 36, 42]. An increase in amplitude of the negative peak at approximately 400-ms post-stimulus onset (N400) for non-rhyming words relative to rhyming words has been observed across rhyming studies. The difference between the N400 elicited by the non-rhyming and rhyming words is called the “rhyme effect.” The rhyme effect is a broadly distributed negative component observed over bilateral central-parietal electrode sites [36]. It is considered to be a part of the family of N400-like potentials that are sensitive to contextual information and underlies the cognitive processes mediating comparison of phonological representation of words [35]. Coch et al. [36] also reported a reversal of the rhyme effect at bilateral anterior electrode sites, the anterior reverse rhyme effect, signaled by a larger amplitude N400 elicited by rhyming targets compared to non-rhyming targets. Spatiotemporal analysis in adults and children performing visual rhyme judgment suggests that the anterior reverse rhyme effect may be specific to the rhyming stimuli and is thought to reflect a cognitive process separate from that elicited by the non-rhyming stimuli at central-parietal sites [43]. We hypothesized that CWNS, CWS-Rec, and CWS-Per would elicit the classic central-parietal rhyme effect, based on previous findings that the N400 for rhyme target processing did not distinguish CWNS and CWS in a more cognitively challenging rhyming task than the one used in the present study [21]. Due to limited discussion regarding the anterior reverse rhyme effect in previous work, we could not make specific predictions about how the distribution of the N400 in anterior electrode sites might distinguish the groups. But based on the findings from Chang et al. [11] and Weber-Fox et al. [21], we predicted that there would be differences in hemispheric contributions for rhyme processing between the CWS-Per and the other two groups.

## Methods

### Participants

The current study was part of a larger longitudinal study examining language and motor factors involved in stuttering. Data were collected at two testing sites—Purdue University and the University of Iowa. For the purpose of this study, 43 children with ages ranging from 6;11 (years;months) to 8;11 served as participants (see Table 1 for subject characteristics). Twenty-two children were judged to be normally fluent (hereafter, CWNS). Twenty-one children had been diagnosed as stuttering at the age of 4 to 5 years (hereafter, CWS). To be considered stuttering, participants had to meet the criteria proposed by Yairi and Ambrose [44]. Specifically, the children were perceptually judged to display stuttering by a parent as well as a speech-language pathologist who worked on the project. The parent and clinician severity ratings were 2 or greater on an eight-point (0–7) stuttering severity scale, with 0 equivalent to no stuttering and 7 equivalent to the greatest severity of stuttering. All children classified as CWS also displayed at least three stuttering-like disfluencies (SLDs) per 100 syllables during a spontaneous language sample. SLDs included part-word repetitions, sound prolongations, and dysrhythmic phonations, including silent blocks.

**Table 1:** Subject characteristics of CWNS, CWS-Rec, and CWS-Per and standard scores on cognitive and language tests

Test	CWNS		CWS-Rec		CWS-Per			
	(*n* = 22)		(*n* = 12)		(*n* = 9)			
	Mean	SE	Mean	SE	Mean	SE	F(2, 43)	*p*
Age	7;6	0.11	7;8	0.15	7;6	0.17		
Sex	15M, 7F	–	10M, 2F	–	7M, 2F	–		
Handedness	18R, 4L	–	10R, 2L	–	9R, 0L	–		
CMMS	111.77	2.23	109.75	2.76	111.11	4.10	0.141	0.869
TACL-3	121.73	2.50	114.83	3.76	113.67	5.91	1.637	0.207
SPELT-3	110.82	1.69	103.67	2.81	103.56	4.20	3.070	0.058
BBTOP-CI	103.23	1.48	94	2.77	94.88	2.52	7.663	0.003*

CMMS Columbia Mental Maturity Scale [47], TACL-3 Test for Auditory Comprehension of Language—third edition [48], SPELT-3 Structured Photographic Expressive Language Test—third edition [49], BBTOP-CI Bankson and Bernthal Test of Phonology—consonant inventory subtest [29], * indicates a statistically significant p-value

Of the 21 children originally classified as CWS, 12 had recovered from stuttering (CWS-Rec) at the time of testing for the current study. Recovery was defined as receiving a rating of 0 or 1 by both parent and clinician on the eight-point stuttering severity measure and displaying fewer than three SLDs per 100 syllables during spontaneous speech. The remaining nine CWS persisted in stuttering (CWS-Per) at the time of testing. The three groups, CWNS, CWS-Rec, and CWS-Per, did not differ in age, *F* (2, 40) = 1.52, *p* = 0.24 or socio-economic status as defined by mother’s highest level of education [45]; *F* (2, 40) = 0.85, *p* = 0.44).

### Screening procedures

All the participants were native English speakers with no reported neurological, language, reading, visual, or hearing impairments. Their hearing was screened at 250, 500, 1000, 2000, 4000, 6000, and 8000 Hz at 20dBHL and found to be bilaterally normal. None of the children displayed impairments of social interaction or restriction of activities as indexed by the Childhood Autism Rating Scale [46].

All the children were also tested on a comprehensive battery to measure non-verbal intelligence and language and phonological abilities. The behavioral test protocol included measurement of non-verbal intelligence using the Columbia Mental Maturity Scale (CMMS) [47], the Test for Auditory Comprehension of Language—third edition (TACL-3) [48] to assess language comprehension, the Structured Photographic Expressive Language Test—third edition (SPELT-3) [49] to examine expressive language abilities including use of morphemes, and the Bankson and Bernthal Test of Phonology—consonant inventory subtest (BBTOP-CI) [29] to measure articulation abilities. All the children displayed normal non-verbal intelligence (CMMS > 94) and normal receptive language (TACL-3 > 91). Standard scores for expressive language (SPELT-3) and phonological abilities (BBTOP-CI) were all within one standard deviation of the mean (see Table 1 for a summary of performance for the three groups).

### Stimuli for rhyme judgment task

The pseudo-word auditory stimuli for eliciting ERPs were taken from the rhyming study by Coch and colleagues [36]. These included 44 pairs of rhyming pseudo-words (e.g., feap-neap, trum-pum) and 44 pairs of non-rhyming pseudo-words (e.g., bry-pag, mag-yare). Each pseudo-word followed phonological rules of English and had no semantic content. The first pseudo-word of the pair was the “prime” and the second pseudo-word was the “target.” The prime-target pairs in the non-rhyming list were made by associating the prime of one rhyming pair with the target of another rhyming pair. As in Coch et al. [36], there were two lists of pseudo-word pairs. A pseudo-word occurred as a target only in one list. The two lists were counterbalanced across participants. A female, native American-English speaker from the authors’ lab produced the pseudo-word stimuli, which were digitized using PRAAT [50] software at a rate of 22.5 kHz. The average pseudo-word duration was 582.4 ms (SD = 72.1).

### Electrophysiological recording

An elastic electrode cap (Quick-cap) containing 32 Ag-Cl electrodes was used to record electrical activity from the scalp. The cap was fitted snugly over the head with the electrodes positioned in homologous locations over the left and right hemispheres, consistent with the international 10-10 system (American Electroencephalographic Society, 1994). The electrode channels included lateral sites F7/F8, FT7/FT8, TP7/TP8, P7/P8; medial sites FP1/FP2, F3/F4, FC3/FC4, CP3/CP4, P3/P4, O1/O2; and midline sites FZ, FCZ, CZ, CPZ, PZ, and OZ. Electrodes were also placed on the left superior and inferior orbital ridge to monitor vertical eye movements. Horizontal movements were monitored by electrodes on the left and right outer canthi. Electrodes on left and right mastoids served as linked references. The electroencephalogram (EEG) signals were amplified using a band pass filter between 0.1 and 100 Hz and digitized online at the rate of 500 samples per second.

### Procedure

ERPs were recorded as a measure of neural processes mediating the rhyming and non-rhyming judgment task. The electrode cap was fitted on the child while he/she played video games or watched a movie. The scalp impedances were adjusted to 5 kΩ or less, after which the child was seated in a sound-attenuating booth. The following instructions were then provided:

- Now you will listen to pretend words. Sometimes the words will rhyme, like “zoof” and “noof”. Sometimes they won’t rhyme, like “jat” and “misk”. Listen to the pairs of words and try to tell if they rhyme or if they don’t rhyme. It is important that you sit very still. While you are listening to the pretend words, look at the mark on the screen in front of you. If you think the words rhyme, press the green button and if they don’t, press the red button. Every once in a while you will see a picture pop up. This is the time when you can move! Every time you see a picture, you can play a turn of our game. When you finish the game, you will get a surprise! We will start now. Remember to listen carefully to the words that rhyme and don’t rhyme. Let’s practice listening to a few now. Are you ready?

The child sat in a sound-treated booth 160 cm away from a 47.5-cm computer monitor. A researcher was present in the booth throughout the experiment to provide support and reinforcement. The stimuli were presented using Presentation® Version 14.9 via a speaker placed directly above the monitor at a level of 70–75 dB SPL. The child was asked to start the presentation of stimuli by pressing any button on a response pad. After a delay of 1080 ms, the prime was presented, followed by an inter-stimulus interval of 1070 ms and then the target. The child was required to make rhyming judgments using the response pad. The use of the left and right buttons for the “yes” and “no” responses were counterbalanced across subjects.

The stimuli were presented in 11 blocks of eight pairs each, with breaks in between. The breaks were indicated to the child by a picture on the computer monitor. During these breaks, a single turn of games such as “Connect 4,” “Tic Tac Toe,” or “Guess Who” was played by the child and researcher.

### EEG data analysis

The three groups, CWNS, CWS-Rec, and CWS-Per, were compared for measures of neural activity in the rhyme judgment task. The data were analyzed using EEGLAB and ERPLAB [51], toolboxes of MATLAB®, (MathWorks, Natick, MA, USA). Only ERP data corresponding to correct responses were analyzed. Eye artifacts were removed from the EEG signals using independent component analysis (ICA; EEGLAB). ICA is an EEGLAB tool that separates independent sources of EEG signals. Artifacts to be removed such as blinks, horizontal eye movements and voltage drifts were identified independently by two trained research assistants. Any discrepancies were resolved by a third independent research assistant. Such components were removed from the continuous EEG data. The EEG signals were low-pass filtered at 30 Hz with a 12-dB roll-off to eliminate high-frequency noise. The continuous EEG records were epoched from 100 ms prior to stimulus onset and continued until 1000 ms post-stimulus for purposes of averaging and ERP component measures. Automatic voltage-dependent artifact removal was then performed on all the EEG channels. The EEG epochs were averaged for the ERPs elicited by the prime, rhyming targets, and non-rhyming targets. The average percentage of usable trials following artifact rejection across the conditions was 80.08 % (SD 9.46) for the primes, 82.27 % (SD 9.7) for the rhyme targets, and 82.05 % (SD 9.99) for the non-rhyme targets. There was no significant difference between groups in the number of trials rejected for any of the conditions, *F* (2, 31) = 0.97, *p* = 0.38.

### Statistical analysis

Percent accuracy of response in judging rhyming from non-rhyming pairs was obtained from the button press responses and a repeated measures ANOVA was used to compare the percentage correct responses for the three groups (CWNS, CWS-Rec, and CWS-Per) across two conditions (rhyme and non-rhyme).

ERP measurements considered for statistical comparison were the peak latencies and mean amplitudes of the N400 ERP component, consistent with previous studies [36]. The peak latency and mean amplitude of the N400 elicited by the prime (N400_P_) and the N400 elicited by the target stimuli (N400_T_) were measured within a 450–700-ms time window. From visual inspection of the grand average ERPs, differences in the onset of the N400_T_ elicited by the rhyming and non-rhyming targets were apparent, particularly over anterior electrode sites. Therefore, the mean amplitude of N400_T_ was also measured within an earlier temporal window of 300–500 ms to capture the onset interval of the component.

Similar to previous ERP studies, e.g., [12, 20, 21, 33, 35, 42], mixed-effects repeated measures analysis of variance (ANOVA) was used for the ERP measures separately for the primes and targets, which included a between-subject factor of group (CWNS, CWS-Rec, and CWS-Per). Within-subject factors for primes were hemisphere (left, right), anterior-posterior (AP) distribution (anterior: frontal and frontal-central; posterior: central, central-parietal, parietal), and laterality (lateral, medial). Within-subject factors for targets included condition (rhyme targets, non-rhyme targets), hemisphere, AP distribution, and laterality. For analysis of the midline electrode sites, a separate mixed-effects repeated measures ANOVA was calculated for primes by AP distribution (anterior: FZ and FCZ; posterior: CZ, CPZ, and PZ) and for targets with condition and AP distribution as within-subject factors. Hyunh-Feldt (H-F) adjusted *p* values were used to determine significance with a criterion of *p* < 0.05. The effect sizes determining strength of associations, indexed by the partial eta squared statistic (*ƞ*^2^_*p*_), are reported for all significant effects. Step-down repeated measures ANOVAs were performed to further explore significant interactions. All the EEG recording, ERP analyses, and statistical measures were consistent with the guidelines specified by Picton et al. [52].

## Results

### Response judgment accuracy

As displayed in Table 2, all three groups were highly accurate in making rhyme judgments, but CWNS performed with slightly higher accuracy rates than the other two groups, *F* (2, 40) = 3.89, H-F *p* = 0.02, *ƞ*^2^_*p*_ = 0.16. All three groups were more accurate in making rhyme judgments for non-rhyming pairs (94.6 %) than rhyming pairs (90.6 %), *F* (1, 40) = 8.16, H-F *p* < 0.01, *ƞ*^2^_*p*_ = 0.17.

**Table 2:** Mean rhyme judgment accuracy for CWNS, CWS-Rec, and CWS-Per

	CWNS		CWS-Rec		CWS-Per	
	Mean (%)	SE	Mean (%)	SE	Mean (%)	SE
Rhyming pairs	94	1.07	88.25	2.98	89.4	1.69
Non-rhyming pairs	96.5	1.29	92.9	1.80	94.2	1.86

### Event-related potentials

#### ERPs elicited by prime pseudo-words

##### N400_P_ mean amplitude

The overall mean amplitudes of the N400_P_ did not differentiate groups, *F* (2, 40) = 0.356, H-F *p* = 0.70, nor were there any interactions with group, *F* (8, 160) < 1, H-F *p* > 0.42. A significant anterior-posterior effect at the lateral/medial sites, *F* (4, 160) = 29.48, H-F *p* < 0.001, *ƞ*^2^_*p*_ = 0.42 and midlines, *F* (5, 200) = 25.73, H-F *p* < 0.001, *ƞ*^2^_*p*_ = 0.391, along with a significant anterior-posterior × laterality effect, *F* (4, 160) = 17.72, H-F *p* = 0.01, *ƞ*^2^_*p*_ = 0.09, revealed that the mean amplitude of the N400_P_ elicited by the primes was largest primarily at the anterior medial sites.

##### N400_P_ peak latency

Based on the distribution of N400_P_, the peak latency of the N400_P_ was measured at six anterior electrode sites: F3/4, FZ/FCZ, and FC3/4. The peak latency measure of the N400_P_ did not differentiate groups, *F* (2, 40) = 0.86, H-F *p* = 0.43, nor were there any group interactions, *F* (10, 200) < 1, H-F *p* > 0.40.

#### ERPs elicited by target pseudo-words

**Fig. 1:**
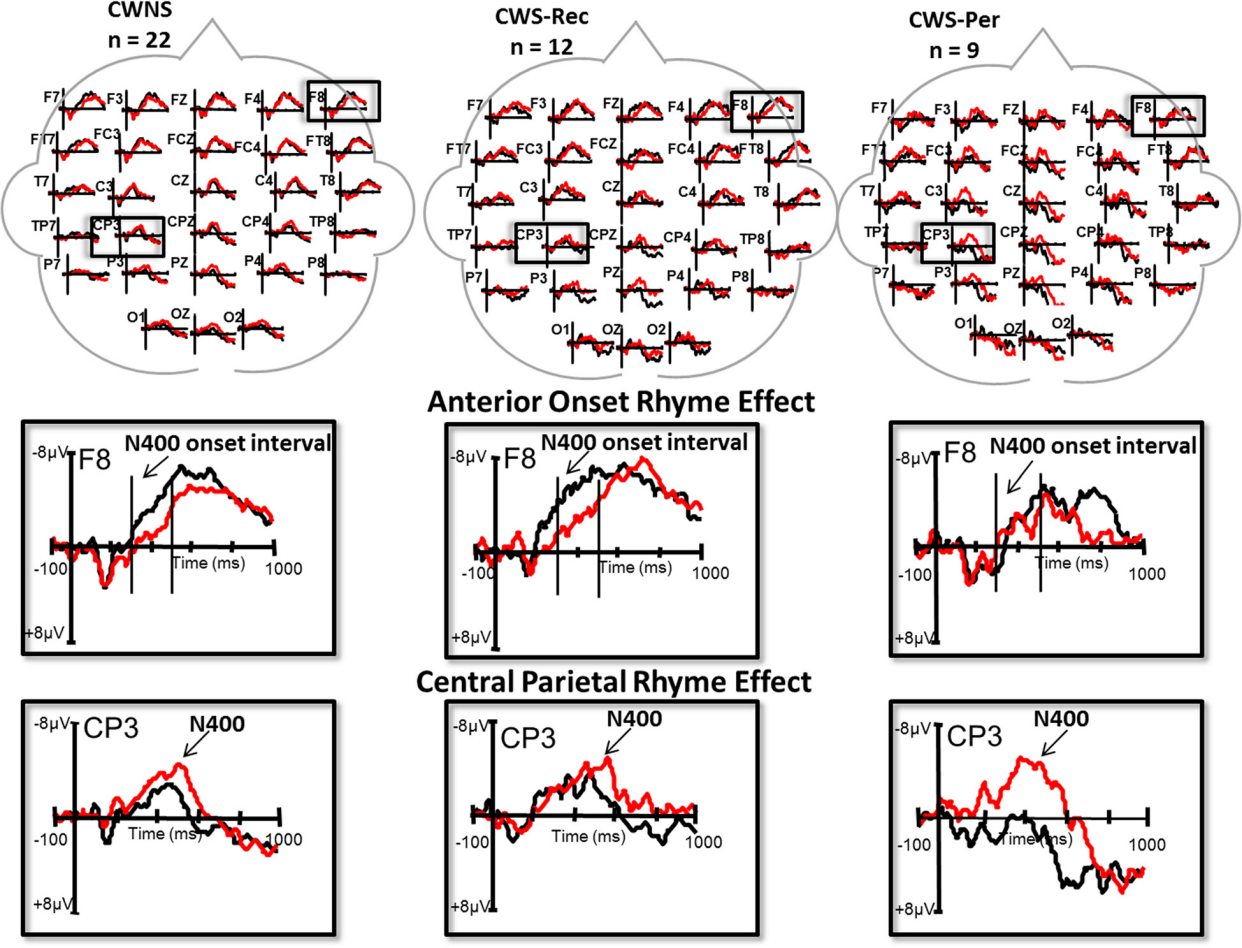
N400_T_ rhyme effect. *Top panel*—grand average ERPs of all participants in CWNS, CWS-Rec, and CWS-Per groups, showing the N400 elicited by the non-rhyming (*red*) and rhyming (*black*) conditions. *Middle panel*—enlarged grand averages of the anterior onset rhyme effect are shown for the right anterior electrode site F8 for the CWNS, CWS-Rec, and CWS-Per groups. *Bottom panel*—enlarged grand averages of the rhyme effect are shown for the left central-parietal electrode site CP3 for the CWNS, CWS-Rec, and CWS-Per groups. Axes scales are provided in the enlarged boxes

The grand average waveforms elicited by the rhyming and non-rhyming target pseudo-words for the CWNS, CWS-Rec, and CWS-Per groups are illustrated in the top panel of Fig. 1.

##### Onset interval of N400_T_

**Fig. 2:**
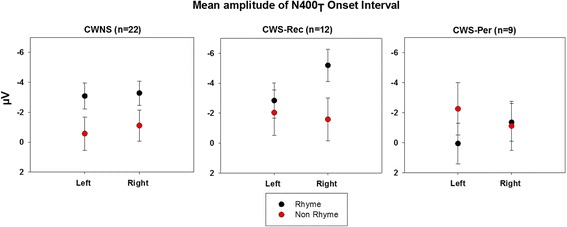
Hemispheric differences in mean amplitude of the N400_T_ onset interval. Mean amplitudes of the N400_T_ onset interval elicited by target stimuli (rhyme vs. non-rhyme) are shown for the left anterior lateral sites (F7/FT7) and right anterior lateral sites (F8/FT8) for the CWNS, CWS-Rec, and CWS-Per groups

The mean amplitude of the N400_T_ during the onset interval was larger for the rhyming targets relative to the non-rhyming targets at anterior lateral sites, condition × AP × laterality *F* (4, 160) = 8.15, H-F *p* < 0.001, *ƞ*^2^_*p*_ = 0.16, hereafter referred to as the anterior onset rhyme effect. A step-down ANOVA was performed in order to explore this interaction at the anterior lateral sites (F7/FT7 and F8/FT8). This revealed a significant group × condition × hemisphere interaction *F* (2, 40) = 3.17, H-F *p* = 0.05, *ƞ*^2^_*p*_ = 0.13. A further step-down ANOVA was done comparing the difference between the ERPs elicited by the rhyming and non-rhyming conditions for each group in each hemisphere, the results of which are illustrated in Fig. 2. The rhyming targets elicited a larger N400 than the non-rhyming targets during this onset interval (300–500 ms) bilaterally for the CWNS; right: *F* (1, 21) = 8.49, H-F *p* < 0.01, *ƞ*^2^_*p*_ = 0.29; left: *F* (1, 21) = 7.08, H-F *p* = 0.02, *ƞ*^2^_*p*_ = 0.25. A trend (*p* = 0.06) indicated a larger mean amplitude of the onset interval of the N400_T_ elicited by the rhyming targets relative to the non-rhyming targets over the right hemisphere for the CWS-Rec, *F* (1, 11) = 4.29, H-F *p* = 0.06, *ƞ*^*2*^_*p*_ = 0.28, but not over the left hemisphere, *F* (1, 11) = 0.34, H-F *p* = 0.57, *ƞ*^2^_*p*_ = 0.03. Given a *p* value of 0.06 in light of the small sample size (*n* = 12) in the CWS-Rec group and low power of the effect (observed power = 0.47) for the step-down ANOVA over the right hemisphere, we were sensitive to the likelihood of making a type II error [53]. Therefore, we inspected the mean amplitude measures of the individual ERP waveforms for each of the CWS-Rec participants. Nine of the 12 CWS-Rec displayed greater mean amplitude measures in the onset interval for the ERPs elicited by the rhyming targets relative to the non-rhyming targets over the right anterior lateral electrode sites. This effect is apparent in the grand average waveforms (Fig. 1) of the CWS-Rec as well as in Fig. 2. In contrast to the anterior onset effect observed in the CWNS (both hemispheres) and CWS-Rec (right hemisphere), the ERPs of the CWS-Per group displayed an opposite pattern—greater mean amplitude onset interval for non-rhyming targets relative to rhyming targets—over the left hemisphere *F* (1, 8) = 11.07, H-F *p* = 0.01, *ƞ*^*2*^_*p*_ = 0.58 (see Fig. 2). There was no difference between conditions over the right hemisphere for the CWS-Per, *F* (1, 8) = 0.04, H-F *p* = 0.85 *ƞ*^2^_*p*_ = 0.01. Visual inspection of individual waveforms of the participants in the CWS-Per group and comparison of mean amplitude values elicited by rhyming and non-rhyming targets over the anterior lateral sites revealed a reversal of the expected pattern over left hemisphere in seven of the nine CWS-Per.

Because of the small sample size and large variability in mean amplitude values between subjects, we confirmed our repeated measures ANOVA results by evaluating the effect of experimental condition using a mixed-effects model (STATA 13 statistical analyses tool), with a random effect for the between-subject variance. Mixed-effects models have been effective in other ERP studies (e.g., [54]). The mixed-effects model was estimated separately for the three groups (CWNS, CWS-Rec, CWS-Per). Each participant had four repeated measures estimated per hemisphere (left, right): two conditions (rhyming, non-rhyming) and two electrode locations (frontal, frontal-temporal). In total, six models were estimated that included the random intercept term measuring inter-individual heterogeneity in mean amplitude value and the residual term. A condition × electrode interaction was tested for each model and found to be not statistically significant in each case (smallest *p* = .62). Therefore, the interaction was excluded from the models. The *β* coefficient provides a test and effect size for the impact of rhyming versus non-rhyming after controlling for electrode location. Marginal mean estimates of mean amplitude value were obtained from the models for rhyming and non-rhyming conditions. Tables 3, 4, and 5 display the results of this mixed-effects model.

**Table 3:** Mixed-model results for CWNS (88 observations for 22 participants)

	Left hemisphere		Right hemisphere	
	*β*	*p* value	*β*	*p* value
Condition (rhyming, non-rhyming)	2.51	0.000	2.17	0.000
Electrode (frontal, frontal-temporal)	0.43	0.485	1.06	0.064
	σ^2^	95 % CI	σ^2^	95 % CI
Random intercept	11.52	(5.71, 23.23)	9.45	(4.67, 19.14)
Residual	8.44	(6.00, 11.87)	7.15	(5.08, 10.06)

**Table 4:** Mixed-model results for CWS-Rec (48 observations for 12 participants)

	Left hemisphere		Right hemisphere	
	*β*	*p* value	*β*	*p* value
Condition (rhyming, non-rhyming)	0.81	0.317	3.61	0.000
Electrode (frontal, frontal-temporal)	1.04	0.199	1.68	0.099
	σ^2^	95 % CI	σ^2^	95 % CI
Random intercept	5.37	(1.78, 16.20)	8.87	(2.98, 26.39)
Residual	7.81	(4.92, 12.40)	12.35	(7.78, 19.60)

**Table 5:** Mixed-model results for CWS-Per (36 observations for 9 participants)

	Left hemisphere		Right hemisphere	
	β	*p*-value	β	*p*-value
Condition (rhyming, non-rhyming)	−2.31	0.009	0.24	0.762
Electrode (frontal, frontal-temporal)	−2.10	0.017	0.63	0.422
	σ^2^	95 % CI	σ^2^	95 % CI
Random intercept	27.32	(10.21, 73.08)	11.5	(4.08, 32.45)
Residual	7.03	(4.12, 11.99)	5.55	(3.26, 9.47)

Mean estimates were the same using the mixed-effects approach as with the repeated measures ANOVA approach. However, the *p* values differed slightly due to the fact that the mixed model did not make strict assumptions about the homogeneity of variance, compound symmetry, or sphericity because the covariance structure was explicitly modeled. Using this approach, our results were replicated, except that *p* values were lower for all the tests. The result of the mixed-effects model indicated a difference between experimental conditions for the CWS-Rec group over the right hemisphere with the *p* < 0.001.

As displayed in Table 1, the BBTOP-CI revealed higher scores for the CWNS as compared to the other two groups. Because the CWS-Rec and CWS-Per performed similarly, it is unlikely that scores on the BBTOP-CI influenced our main finding that the anterior onset rhyme effect distinguished CWS-Per from the other two groups. In order to confirm this assumption, a Pearson correlation was performed to assess whether the differences between groups for the mean amplitude onset interval N400_T_ at the anterior sites were associated with differences in the BBTOP-CI scores. The BBTOP-CI scores for the participants were not correlated with the mean amplitude onset interval of the N400_T_ for the left hemisphere, *r* = −2.16, *p* = 0.16, or for the right hemisphere, *r* = 0.03, *p* = 0.84.

##### N400_T_ mean amplitude

The N400_T_ mean amplitude for the non-rhyming targets was more negative as compared to the N400_T_ amplitude for the rhyming targets over central-parietal electrode sites (CP3, CPZ, CP4, P3, PZ, P4; classic rhyme effect) for all the three groups, *F* (4, 160) = 4.89, H-F *p* < 0.01, *ƞ*^2^_*p*_ *=* 0.16 (lower panel of Fig. 1). There were no interactions with condition, *F* (1, 40) < 2.33, H-F *p* > 0.13 or group interactions, *F* (8, 160) < 1, H-F *p* > 0.58.

##### N400_T_ peak latency

There were no condition effects across lateral/medial electrode sites, *F* (1, 40) < 3.51, H-F *p* > 0.07 or across midlines, F (1, 40) < 1, H-F *p* > 0.35, nor were there any significant interactions with group at lateral/medial sites, *F* (8, 160) < 1, H-F *p* > 0.48 or midlines, *F* (10, 200) < 1, H-F *p* > 0.34.

## Discussion

Using a rhyming paradigm, we asked whether the neural indices of phonological processing would differentiate 7–8-year-old typically fluent children, those who had recovered from stuttering and those whose stuttering persisted. Results indicated that although all three groups made accurate overt rhyme judgments, there were differences in the underlying neural activity that mediated the processing of the rhyming and non-rhyming targets, as evidenced by the ERPs. All three groups demonstrated the classic central-parietal rhyme effect. However, the anterior onset rhyme effect was absent in the children with persistent stuttering. Further, the distribution of the anterior effect, while bilateral in the CWNS group, was most pronounced over the right hemisphere for the CWS-Rec.

The use of strict inclusionary criteria ensured that the groups were matched for age, gender distribution, handedness, socio-economic status, non-verbal IQ (CMMS), language comprehension (TACL-3), and language expression (SPELT-3). The CWS-Rec and CWS-Per performed similarly on the test of articulation abilities, the BBTOP-CI. But the CWNS had scores that were slightly higher than the CWS on this test. Follow-up analyses, however, revealed that the differences in BBTOP-CI scores across groups did not account for the group differences in the mean amplitudes of the onset interval of the N400 elicited by the targets.

### Behavioral accuracy

Children in all three groups were more accurate in making non-rhyme decisions compared to rhyme decisions, consistent with results from Coch et al. [36]. Although the typically fluent children performed with slightly greater accuracy in the rhyme judgment task, it was evident that the children in all three groups were highly proficient in making rhyme judgments. In a previous study, CWS and CWNS in this age range also showed high proficiency for other phonological tasks such as rhyme monitoring [55]. However, Weber-Fox et al. [21] found that older school-age CWS (9–13 years) had significantly lower rhyme judgment accuracy compared to the typically fluent children in a *visual* rhyming task that involved orthographic constraints. The additional demand of orthographic incongruence as well as the requirement to respond as quickly as possible likely enhanced the differences in behavioral accuracy between the groups. The task in our study was significantly different—it was in the auditory modality, relatively simple, and resulted in significant but smaller differences in rhyme judgment accuracy between the CWS (Rec and Per) and CWNS. It is important to note, however, that the CWS-Rec and CWS-Per did not differ in their behavioral accuracy, so this cannot account for any differences in the neural indices of phonological processing observed between these two groups.

### N400_T_ rhyme effect

The well-known central-parietal rhyme effect, indexed by larger N400_T_ mean amplitude elicited by non-rhyming stimuli relative to rhyming stimuli, was observed in all three groups as predicted. Weber-Fox et al. [21] also observed a rhyme effect in their visual rhyming study in 9–13 year old CWS and fluent peers. The rhyme effect is thought to index neural processes mediating the comparison of phonological representations of words [34, 35]. Thus, in the current study, it seems likely that the neural functions that mediate the comparison and ease of integration of phonological properties of the prime and target were similar for CWNS, CWS-Rec, and CWS-Per.

### N400_T_ onset interval

The anterior effect observed in our study refers to a measure of the mean amplitude of the *onset* interval of the N400_T_ measured in the 300–500 ms temporal window. In earlier studies, the rhyme and reverse rhyme effects were elicited concurrently [36, 42], and the anterior effect was considered to be a “reversal” of the central-parietal rhyme effect. We believe that this interpretation may be inadequate. The anterior onset rhyme effect and central-parietal rhyme effect may function independently to some degree. Findings from Khateb and colleagues [43] indicated that adults’ ERPs elicited by the rhyming condition relative to non-rhyming condition started before 300 ms post-stimulus onset and peaked at 350 ms at left frontal-temporal sites, while the central-parietal rhyme effect (non-rhyme > rhyme) maximum amplitude occurred at 400 ms. Khateb and colleagues suggested that the anterior reverse effect and the central-parietal rhyme effect involve distinct neural processes. Our findings indicated group differences for the anterior, but not central-parietal rhyme effect, providing further evidence that these component indices may be distinct. Taken together, with earlier findings [43], our results suggest that the N400_T_ onset over anterior lateral electrodes may index an earlier phonological processing stage separate from the phonological integration stage indexed by the N400_T_ elicited over central-parietal sites.

The rhyme judgment task using prime-target pseudo-word pairs utilized in the present study involved automatic processes including segmenting the prime into its rime component, holding it briefly in verbal working memory, and then comparing the rime with the upcoming target. This task involved both phonological segmentation and rehearsal, functions that have been found to involve Broca’s area and the precentral gyrus [56]. In such a task, the phonologically related prime pre-activates some of the targets’ segments ([36, 57], Expt 2) and hence, target processing in the rhyming condition occurs with greater efficiency. While efficient processing of rhyming targets is represented as smaller amplitude N400_T_ as compared to non-rhyming targets over central-parietal electrode sites during phonological integration, a different pattern was exhibited over anterior lateral sites. We speculate that over anterior lateral sites, prime facilitation and subsequently, more efficient processing of rhyming targets may have been indexed by earlier onset of N400_T_ for rhyming relative to non-rhyming targets.

Comparable peak latencies of the N400 elicited by the primes indicated that processing of the phonological components of the primes was similar across all three groups. However, the anterior onset rhyme effect distinguished the groups, as demonstrated by the significant group × condition × hemisphere interaction. Based on converging evidence from three prior studies [36, 42, 43] and the current findings for the anterior rhyme effect, we interpreted the presence of an anterior onset rhyme effect in the CWNS and CWS-Rec groups as reflecting a facilitation for processing the rhyming targets relative to the non-rhyming targets. These findings suggest that the representation of the prime for the CWNS and CWS-Rec groups was likely more salient and robust in these groups compared to the CWS-Per. For the CWS-Per, it may be that the prime did not facilitate phonological access to the rhyming targets in this early window, exhibited by the absence of the anterior onset rhyme effect. However, a robust central-parietal rhyme effect observed in all the groups indicated sensitivity to phonological mismatch of the rime in the non-rhyming condition. Thus, the rhyming task in the present study elicited group differences in the stage of neural processing involving phonological segmentation and rehearsal but not at the stage indexing phonological integration.

Additionally, there were hemispheric differences between the groups for processing the rhyming target at the anterior electrode sites, as verified by the mixed-effects model. The CWNS displayed prime facilitation for processing the rhyming targets equally in both hemispheres, consistent with the finding of bilateral anterior rhyme effect observed in typically developing 7- and 8-year-olds in Coch et al. [36], while the CWS-Rec did not show this “fluent” pattern. The anterior onset rhyme effect was more predominant over the right hemisphere for the CWS-Rec. Despite the reduced power of the effect, the larger amplitude of the ERPs elicited by the rhyming compared to the non-rhyming targets in the onset interval over the right hemisphere was seen in nine of the 12 CWS-Rec and indicated that for this particular stage of phonological processing, many CWS-Rec recruited hemispheric functions differently as compared to the CWNS. To our knowledge, this is the first study that examines neural activation during phonological processing in children who have recovered from stuttering. Using neuroanatomical imaging, Chang et al. [11] indicated a non-significant trend towards greater white matter fractional anisotropy in certain regions of the brain, including the right rolandic operculum and right supramarginal gyrus in the recovered group. The left and right supramarginal gyri have been shown to be involved in phonological processing, as observed using transcranial magnetic stimulation [58]. However, the link between our electrophysiological findings and the structural imaging data from Chang et al. [11] is unclear, given that EEGs have poor spatial resolution and identifying the location and orientation of neural generators is difficult. Therefore, more neurophysiological evidence is required to evaluate the role of the right hemisphere in the neurodevelopmental course of stuttering recovery.

In contrast to the CWNS and CWS-Rec, the CWS-Per group did not show an anterior onset rhyme effect—an increased amplitude N400_T_ elicited by rhyming target relative to non-rhyming target at anterior sites—in either hemisphere. They did, however, exhibit a reversal of the expected pattern over the left hemisphere. While the significance of the reversed pattern is difficult to interpret at this point in time, the absence of prime facilitation at the anterior electrode sites for the CWS-Per suggests a difference in phonological processing as compared to the other two groups. These findings argue against the domain-specific theories of stuttering in favor of the dynamic interactions model [7]. Stuttering is not just a series of “stutter events”—linguistic, emotional, and motor processes interact in specific ways that influence the probability of speech motor breakdown. Phonological processing and encoding interact very closely with speech production processes. Atypical development of any of these processes could likely be related to breakdown in another domain. Although many children who stutter typically score within normal limits on phonological, semantic, or morphosyntactic tests, it may be conceivable that the presence of subtle communicative difficulties would tax the weaker or less stable speech production system, contributing to a greater likelihood of repeated or prolonged speech sounds. For example, recent ERP evidence indicates that young children (age 6–7 years) whose stuttering is persisting display less mature neural systems for processing of phrase structure violations when presented in Jabberwocky sentences compared to age-matched children who have recovered from stuttering and typically developing peers [12].

Our results indicate that despite comparable non-verbal intelligence, language skills, phonological skills, and behavioral rhyme judgment accuracy, neural activity mediating phonological processing necessary for rhyme judgments may distinguish CWS-Rec from CWS-Per. The children whose stuttering persisted did not show the anterior onset rhyme effect, indicating less efficient rime access and prime facilitation during the stage of phonological segmentation and rehearsal. While heterogeneity of neural correlates of language processing in CWS is typical, an absence of the anterior onset rhyme effect in 78 % (7/9) of the CWS-Per suggested that this may be a valuable indicator of the neurodevelopmental characteristic of stuttering persistence. Additionally, although the target stimuli elicited the anterior onset rhyme effect for the CWS-Rec and the CWNS, hemispheric differences in the neural indices of phonological processing suggested that the CWS-Rec did not process the rhyming target in the same way as the CWNS. The children who had recovered from stuttering showed a tendency for greater right hemisphere involvement during target processing as compared to the typically fluent children who showed bilateral processing. Therefore, the children who had recovered from stuttering still exhibited some traces of a history of stuttering during phonological processing, as evidenced by the electrophysiological measures. A crucial next step is to determine if these neurophysiological differences for phonological processing are evident earlier in development when all the children stutter. These findings also have important implications for how neurodevelopmental trajectories differ for children who stutter compared to typically fluent peers.

### Limitations

Efforts were taken to ensure that the groups were matched for various factors in order to eliminate potential confounding variables. However, some variables that were not controlled were time since stuttering onset, duration of recovery status, and the effect of stuttering therapy. Future studies should examine the possible role these variables may have on the differences in neural functions among children who have recovered from stuttering and those who persist.

In the current study, we only describe the neural indices of phonological processing in groups of children who have already recovered and whose stuttering persisted at the time of the study. It is possible that some of the children who were classified as CWS-Per will recover in the future. However, the likelihood of recovery reduces with increase in years since stuttering onset [2]. Seven out of the nine children in the CWS-Per group had been diagnosed 3 years prior to testing, while two had been diagnosed 2 years prior. Therefore, given the children’s ages and time since stuttering onset, the chances of recovery among CWS-Per in our study are minimal. While we examined young school-aged children, longitudinal assessment of children nearer the age of stuttering onset may prove valuable in identifying an early, predictive neural marker of stuttering recovery and persistence.

The interpretation of the current ERP findings for the prime facilitation (anterior onset rhyme effect) would have been enhanced with reaction time measures. However, because it is difficult to get reliable reaction time measures from 7–8-year-old children, we did not instruct them to respond as quickly as possible during rhyme judgment. On the other hand, the ERP measures provide a means of examining phonological processing and possible priming in young children when behavioral responses may be unreliable in this population.

## Conclusions

Our findings are the first to show that neural activity mediating phonological processing necessary for rhyme judgment may distinguish 7–8-year-old children who have recovered from those whose stuttering persists. Specifically, we suggest the onset interval of the anterior rhyme effect, which likely indexes a different, earlier stage of phonological processing (phonological segmentation and rehearsal) as compared to the central-parietal rhyme effect (phonological integration), is the stage of processing that distinguishes the neural patterns for the children who recovered from those whose stuttering persists. Further investigation in younger children who stutter is necessary to determine if the absence of anterior prime facilitation is an early marker that may help predict which children who stutter are likely to persist.
